# Immunogenic cell death due to a new photodynamic therapy (PDT) with glycoconjugated chlorin (G-chlorin)

**DOI:** 10.18632/oncotarget.9725

**Published:** 2016-05-30

**Authors:** Mamoru Tanaka, Hiromi Kataoka, Shigenobu Yano, Takuya Sawada, Haruo Akashi, Masahiro Inoue, Shugo Suzuki, Yusuke Inagaki, Noriyuki Hayashi, Hirotada Nishie, Takaya Shimura, Tsutomu Mizoshita, Yoshinori Mori, Eiji Kubota, Satoshi Tanida, Satoru Takahashi, Takashi Joh

**Affiliations:** ^1^ Departments of Gastroenterology and Metabolism, Nagoya City University Graduate School of Medical Sciences, Mizuho-cho, Mizuho-ku, Nagoya 467-8601, Japan; ^2^ Graduate School of Materials Science, Nara Institute of Science and Technology, Ikoma, Nara 630-0192, Japan; ^3^ Research Institute of Natural Sciences, Okayama University of Science, Okayama 700-0005, Japan; ^4^ Department of Biochemistry, Osaka Medical Centre for Cancer and Cardiovascular Diseases, Higashinari-ku, Osaka 537-8511, Japan; ^5^ Department of Experimental Pathology and Tumor Biology, Nagoya City University Graduate School of Medical Sciences, Mizuho-cho, Mizuho-ku, Nagoya 467-8601, Japan

**Keywords:** glycoconjugated chlorin (G-chlorin), photodynamic therapy, calreticulin (CRT), high-mobility group box 1 protein (HMGB1), immunogenic cell death (ICD)

## Abstract

Both the pre-apoptotic exposure to calreticulin (CRT) and the post-apoptotic release of high-mobility group box 1 protein (HMGB1) are required for immunogenic cell death. Photodynamic therapy (PDT) uses non-toxic photosensitizers and visible light at a specific wavelength in combination with oxygen to produce cytotoxic reactive oxygen species that kill malignant cells by apoptosis and/or necrosis, shut down the tumor microvasculature, and stimulate the host immune system. We have previously shown that glycoconjugated chlorin (G-chlorin) has superior cancer cell selectivity and effectively suppresses the growth of xenograft tumors. In the present study, we evaluated the immunogenicity of PDT with G-chlorin treatment in colon cancer cells. PDT with G-chlorin suppressed CT26 (mouse colon cancer cells) tumor growth considerably more efficiently in immunocompetent mice (wild-type mice, allograft model) than in immune-deficient mice (nude mice, xenograft model), although control treatments were not different between the two. This treatment also induced CRT translocation and HMGB1 release in cells, as shown by western blot and immunofluorescence staining. To evaluate the use of PDT-treated cells as a tumor vaccine, we employed a syngeneic mouse tumor model (allograft model). Mice inoculated with PDT-treated CT26 cells were significantly protected against a subsequent challenge with live CT26 cells, and this protection was inhibited by siRNA for CRT or HMGB1. In conclusion, PDT with G-chlorin treatment induced immunogenic cell death in a mouse model, where the immunogenicity of this treatment was directed by CRT expression and HMGB1 release.

## INTRODUCTION

Photodynamic therapy (PDT) consists of the administration of a photosensitizer together with visible light irradiation at a specific wavelength to activate the photosensitizer [1], leading to the conversion of molecular oxygen to various highly reactive oxygen species (ROS), which either kill tumor cells directly or damage the tumor-associated vasculature [1, 2]. PDT has several advantages over conventional cancer treatments [3]. First, PDT is relatively non-invasive because of its limited use of irradiation at the tumor site [3]. In addition, PDT shows lower systemic toxicity and a relatively selective destruction of tumors, partly owing to preferential localization of the photosensitizer within the tumor [2]. Thus, PDT has been widely employed to treat various types of tumors that can be directly contacted by irradiation, such as lung carcinomas, esophageal carcinomas, gastric cancer, breast cancer, head and neck tumors, bladder tumors, and prostate tumors [1]. Compared with other therapies, PDT often produces a high cure rate and low recurrence rate [4].

Recently, more and more efforts are addressing the application of particular stress agents that can induce immunogenic cell death (ICD) in cancer cells. One such therapeutic mode associated with ICD is PDT [5–8]. The immunogenic characteristics of ICD are mediated mainly by damage-associated molecular patterns (DAMPs), which include surface-exposed calreticulin (CRT) and release of high-mobility group protein B1 (HMGB1) [9–12].

ICD is preceded by the pre-apoptotic exposure of CRT on the plasma membrane [13]. Although CRT is usually located in the lumen of the endoplasmic reticulum (ER), it translocates to the cell surface during an immunogenic response. This involves a complex signal transduction pathway, which includes an ER stress response, the sub-apoptotic activation of caspase-8, and the exocytosis-dependent co-translocation of CRT, together with another ER protein, ERp57, to the outer surface of the plasma membrane [14]. Surface CRT serves as an engulfment signal and targets apoptotic cells for interaction with Dendritic Cells (DCs), leading to the subsequent cross-presentation of tumor antigens [13, 15].

For immunogenicity to be detected, dying cells must emit signals in addition to CRT. In fact, dying cells release HMGB1 into the extracellular space in response to most chemotherapeutic agents, and neutralization or depletion of HMGB1 abolishes the immunogenicity of cell death [16] [17]. HMGB1 can interact with several receptors expressed on the surface of DCs, including toll like receptor 4 (TLR4) [18].

G-chlorin (glycoconjugated chlorin; glucose-linked tetra (fluorophenyl) chlorin) has been developed as a new type of photosensitizer. We previously reported that G-chlorin-mediated PDT was able to induce apoptosis via singlet oxygen and is about 30 times more cytotoxic than Talaporfin-mediated PDT. Therefore, G-chlorin is a potential photosensitizer of PDT for treating gastric and colon cancer and GIST *in vitro* and *in vivo* [19, 20]. Furthermore, we found that PDT with mannose-conjugated chlorin displayed very strong anticancer effects [21].

In the current study, we show that PDT with a new photosensitizer, G-chlorin, induces ICD by exposure of CRT and release of HMGB1.

## RESULTS

### Antitumor effects of PDT with G-chlorin in immunocompetent or immunodeficient mice

**Figure 1:**
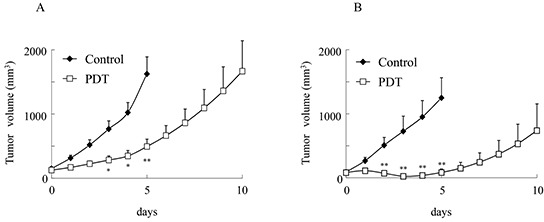
Inhibition of tumor growth of PDT with G-chlorin CT26 cells were inoculated into the dorsal skin of immunocompetent or immunodeficient mice at a concentration of 1 × 10^6^ cells/200 μL in PBS. Tumor-bearing mice were intravenously injected with 6.25 μmol/kg G-chlorin and, after 4 hours, were illuminated with 660-nm LED light (40 J/cm^2^). Each group comprised five mice. Data are mean ± SD. Significance was determined by Welch's *t*-test. * P < 0.05, ** P < 0.01 relative to control. (**A.** immunocompetent mice, **B.** immunodeficient mice)

We examined the effects of G-chlorin-mediated PDT on CT26 tumors in immunocompetent or immunodeficient mice. PDT was performed on the xenograft tumor models in which mouse colon cancer cells (CT26) had been implanted subcutaneously. G-chlorin-mediated PDT suppressed tumor growth substantially in immunocompetent and immunodeficient mice. The suppression was stronger in immunocompetent mice than in immunodeficient mice (Figure 1). All therapies had no obvious side effects, such as diarrhea and weight loss (data not shown). These findings suggest that the immune system may help the antitumor effects of PDT.

### PDT with G-chlorin induced expression of CRT and HMGB1

**Figure 2:**
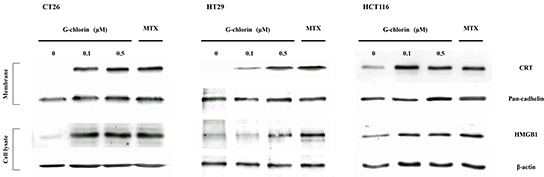
Expression of CRT and HMGB1 by PDT CT26, HT29, or HCT116 cells were loaded with G-chlorin for 4 hours and then irradiated with 16 J/cm^2^ of 660-nm LED light. CT26, HT29, and HCT116 cells were incubated with 1 μM mitoxantrone (MTX) as a positive control. CRT or HMGB1 protein expression was measured by western blotting at 4 hours after treatment.

We examined the expression of CRT and HMGB1 after induction with PDT with G-chlorin *in vitro*. Cells were incubated with mitoxantrone as a positive control. We measured expression of CRT and HMGB1 by western blot. Cell surface proteins in the plasma membrane fraction were tested for CRT. Total cell lysates were also obtained to test for HMGB1. Our results showed that the treatment induced both CRT expression in the plasma membrane fraction and HMGB1 expression in the total cell lysates, not only in mouse colon cancer cells but also in human colon cancer cells (Figure 2). The expression of CRT and HMGB1 mRNA was also increased by PDT with G-chlorin (Supplementary Figure S1).

### PDT with G-chlorin induced translocation of CRT and HMGB1

**Figure 3:**
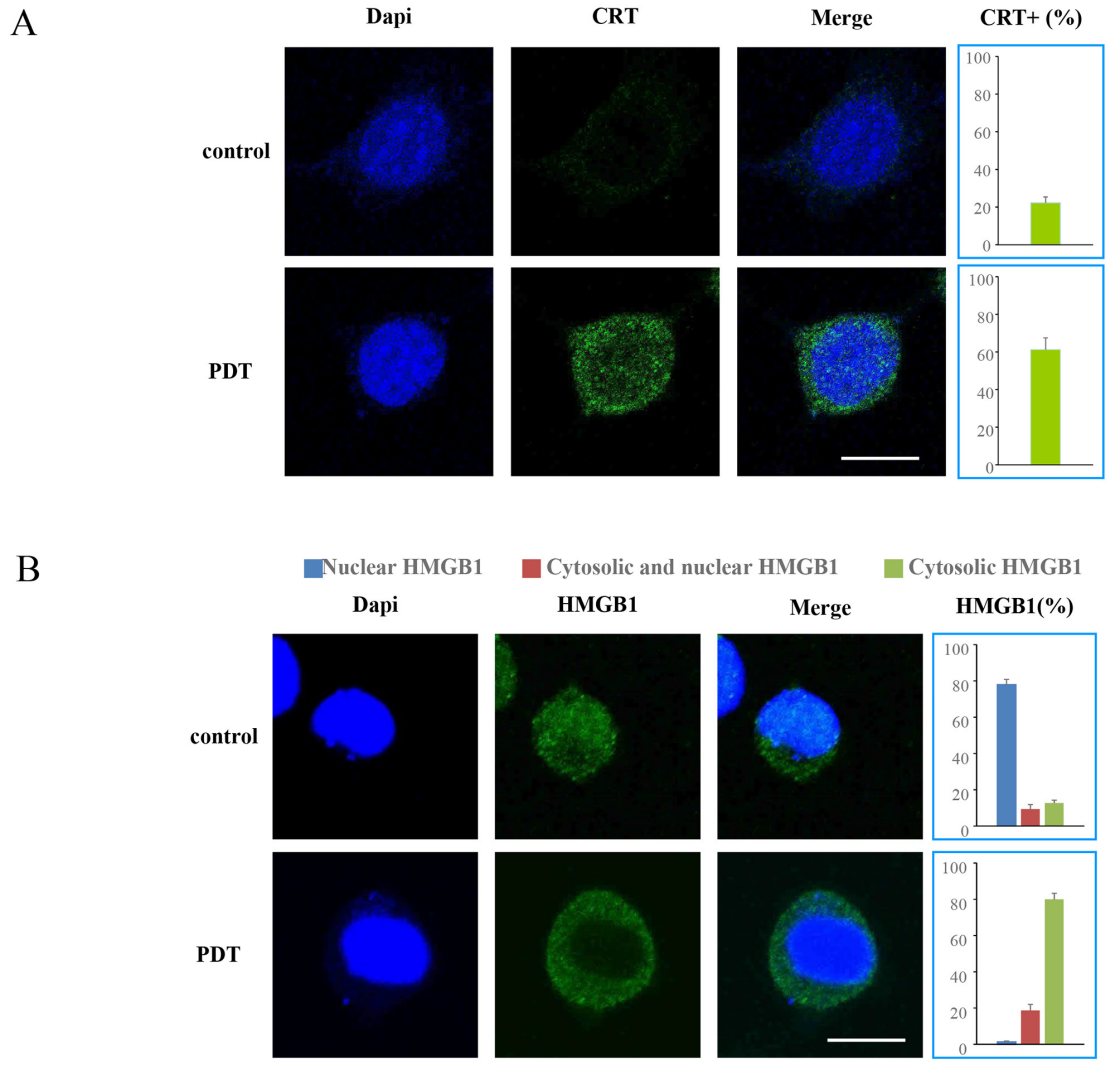
Translocation of CRT and HMGB1 by PDT CT26 cells were loaded with G-chlorin for 4 hours and irradiated with 16 J/cm^2^ of 660-nm LED light. Translocation of CRT and HMGB1 was assessed by immunofluorescence staining at 4 hours after treatment with PDT with G-chlorin. Images were obtained using confocal microscopy (original magnification ×1000; scale bar = 10 μm). Data are means of three independent experiments ± SD. (**A.** CRT, **B.** HMGB1)

We measured the translocation of CRT and HMGB1 after induction with PDT and G-chlorin, using immunofluorescence staining and confocal microscopy. At 4 hours after PDT treatment, an increase in the expression of CRT in the cytoplasm and the release of HMGB1 from the nucleus were induced in CT26 cells (Figure 3). The confocal microscopy images of CRT and HMGB1 translocation at lower magnification is also shown in Supplementary Figure S2.

### PDT with G-chlorin increased the expression of CRT and HMGB1 *in vivo*

**Figure 4:**
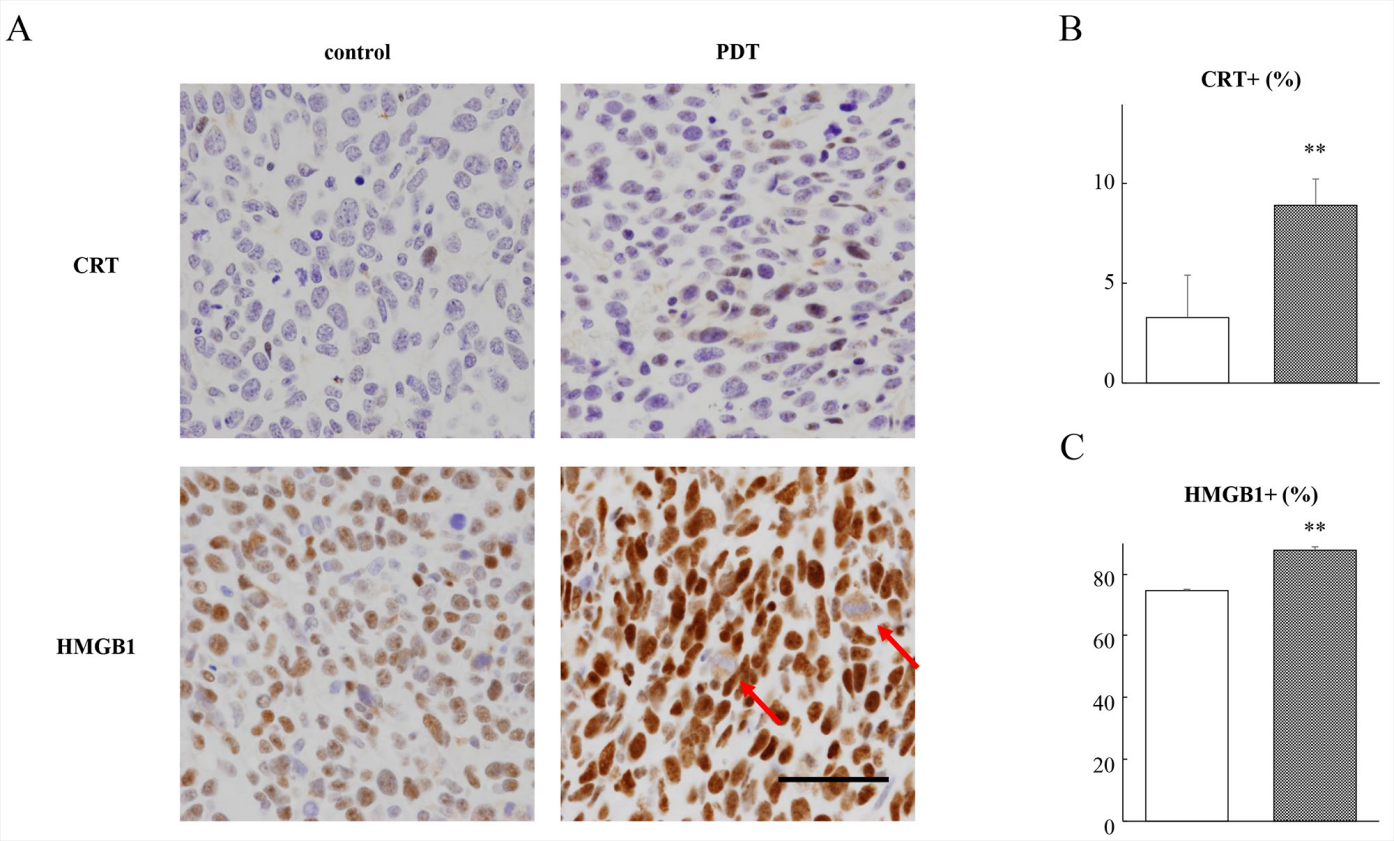
Immunohistochemistry of allograft tumors CT26 cells were inoculated into the dorsal skin of mice. Tumor-bearing mice were intravenously injected with 1.25 μmol/kg G-chlorin and, after 4 hours, were illuminated with 660-nm LED light (15 J/cm^2^). The tumors were excised and fixed in formalin for immunohistochemical examination at 3 hours after treatment with PDT plus G-chlorin. Representative immunohistochemical findings for lesions in the tumors of the control and PDT-treated mice (Panel **A.** original magnification ×400; scale bar = 50 μm). CRT **B.** and HMGB1 **C.** labeling indices in tumors. Data are the mean ± SD. Significance was determined by Welch's *t*-test. ** P < 0.01 relative to control.

After PDT plus G-chlorin treatment of the tumors *in vivo*, the expression levels of CRT and HMGB1 were measured by immunohistochemistry. CRT and HMGB1 were stained in the nucleus of most tumor cells. After PDT treatment, both CRT and HMGB1 were significantly stained in the tumor cells (Figure 4A). Additionally, staining of cytoplasmic HMGB1 was observed in a few tumor cells (Figure 4A, arrows). There was also a significant increase in the labeling index of CRT and HMGB1 in the tumors (Figure 4B, 4C).

### PDT with G-chlorin treated cells vaccinated efficiently *in vivo*

**Figure 5:**
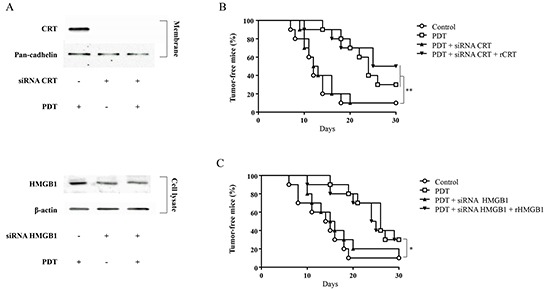
Vaccination **A.** Knockdown of CRT or HMGB1 by using short interfering RNA (siRNA). CT26 cells were transiently transfected with siRNAs against CRT or HMGB1. CRT or HMGB1 protein expression was analyzed by western blotting at 48 hours after transfection. **B, C.** CT26 cells treated *in vitro* with G-chlorin-PDT were inoculated subcutaneously into BALB/c mice. After 7 days, mice were re-challenged with live CT26 cells. The percentages of tumor-free mice were pooled. Each group comprised ten mice. Significance was determined by the log-rank statistic. * P < 0.05, ** P < 0.01 relative to control. (B; CRT, C; HMGB1)

In order to evaluate the use of PDT-treated CT26 cells as a tumor vaccine, we employed a syngeneic mouse tumor model. CT26 cells were loaded with G-chlorin (0.5 μM) for 4 hours, then irradiated with 16 J/cm^2^ of 660 nm LED light. The CT26 cells treated *in vitro* with PDT were inoculated subcutaneously into immunocompetent mice as a vaccine. Seven days later, mice were re-challenged with live CT26 cells. Our results show that PDT with G-chlorin treated CT26 cells vaccinated efficiently against cancer (Figure 5B, 5C).

Next, CT26 cells were transfected with siRNA for CRT or HMGB1, and then treated with PDT *in vitro*. Figure 5A shows the knockdown of CRT and HMGB1 by siRNAs in CT26 cells. Depletion of CRT or HMGB1 with siRNA abolished the immunogenicity of PDT with G-chlorin treated CT26 cells *in vivo* (Figure 5B, 6C). Moreover, when recombinant CRT or HMGB1 (rCRT or rHMGB1) was coated onto the cells before subcutaneous injection, absorbance of rCRT or rHMGB1 restored the lost immunogenicity of CRT- or HMGB1- depleted PDT treated cells (Figure 5B, 5C).

These results indicate that CRT and HMGB1 induction by PDT plays a crucial role in the antitumor effects of PDT, and increases anticancer immunity.

## DISCUSSION

The immunogenicity of dying tumor cells has the ability to drive a strong immune response against cancer cells that have survived therapy. Indeed, in response to anthracyclines (e.g., doxorubicin and mitoxantrone), oxaliplatin, and ionizing irradiation, dying cancer cells trigger tumor-specific immune responses [22–25]. A new concept of immunogenic cell death (ICD) has recently been proposed. ICD is characterized by the secretion, release, or surface exposure of damage-associated molecular patterns (DAMPs) [26]. Several preclinical and clinical studies have demonstrated that PDT activates the host immune response [27, 28]; however, the mechanism of how this happens is not clear.

In this study, we evaluated the immunogenicity of PDT with G-chlorin in colon cancer *in vitro* and *in vivo*. *In vitro*, PDT with G-chlorin induced the translocation of CRT and the release of HMGB1. Mice inoculated with PDT-treated cells were significantly protected against a subsequent challenge with live cells.

The survival rates of cancer patients with metastases decrease significantly compared with those of cancer patients without metastases [29]. There are few effective treatments for metastases, and thus, there is an increased interest in therapies that eliminate primary tumors and systemically activate antitumor immune responses. In the current study, we showed that PDT with G-chlorin suppressed tumor growth in immunocompetent mice than in immunodeficient mice (Figure 1), demonstrating that the immune system may contribute to the therapeutic response of G-chlorin -mediated PDT.

Previous studies have shown that CRT is also expressed in the cytosol and on the cell surface [30, 31]. It has been reported that the KDEL (Lys-Asp-Glu-Leu) ER-retrieval sequence is important for CRT's translocation from the ER lumen to the cytosol and subsequently to the cell surface [32, 33]. CRT plasma membrane exposure act as an “eat me” signal and allow for an optimal anticancer chemotherapy [13, 34]. HMGB1 is released into the extracellular environment during cell death, and this acts as an “alarm” for the innate immune system by acting as a chemoattractant for inflammatory leukocytes, functioning as an immune adjuvant for soluble and particular antigens, and triggering the activation of DCs [36, 36]. In the present study, we demonstrated that G-chlorin-mediated PDT induced CRT membrane exposure and the release of HMGB1 *in vitro* (Figure 2, 3) and *in vivo* (Figure 4). Furthermore, in an antitumor vaccination experiment, PDT with G-chlorin significantly enhanced the inhibitory effects of a tumor cell vaccine on homoplastic grafted tumor growth (Figure 5).

Korbelik et al. reported that PDT with photofrin increased the expression of CRT on the cell surface and the release of HMGB1 in Lewis lung carcinoma (LLC) [37]. In the present study, we demonstrated that PDT with G-chlorin had the same effect, and that these DAMPs were one of the main characteristics of ICD in mice (Figure 5).

PDT with G-chlorin directly kills tumor cells by inducing necrosis and/or apoptosis via ROS production, in such a way that the dying cells expose CRT and release HMGB1, thereby indirectly activating immune effectors. This added immune effect improves the therapeutic efficacy. Our study indicates that the PDT with G-chlorin-induced antitumor immunity could also be used in an adjuvant setting with other local treatments, such as surgery, which is effective at controlling primary tumors without any effect on spreading the disease. Thus, PDT with G-chlorin has the potential to expand the use of this treatment.

In conclusion, we demonstrated that G-chlorin-mediated PDT effectively elicits ICD in colon cancer. The immunogenicity of PDT with G-chlorin-induced cell death is governed by the same rules that apply to those elicited by anthracyclines, in that it involves CRT exposure and HMGB1 release.

## MATERIALS AND METHODS

### Photosensitizers

**Figure 6:**
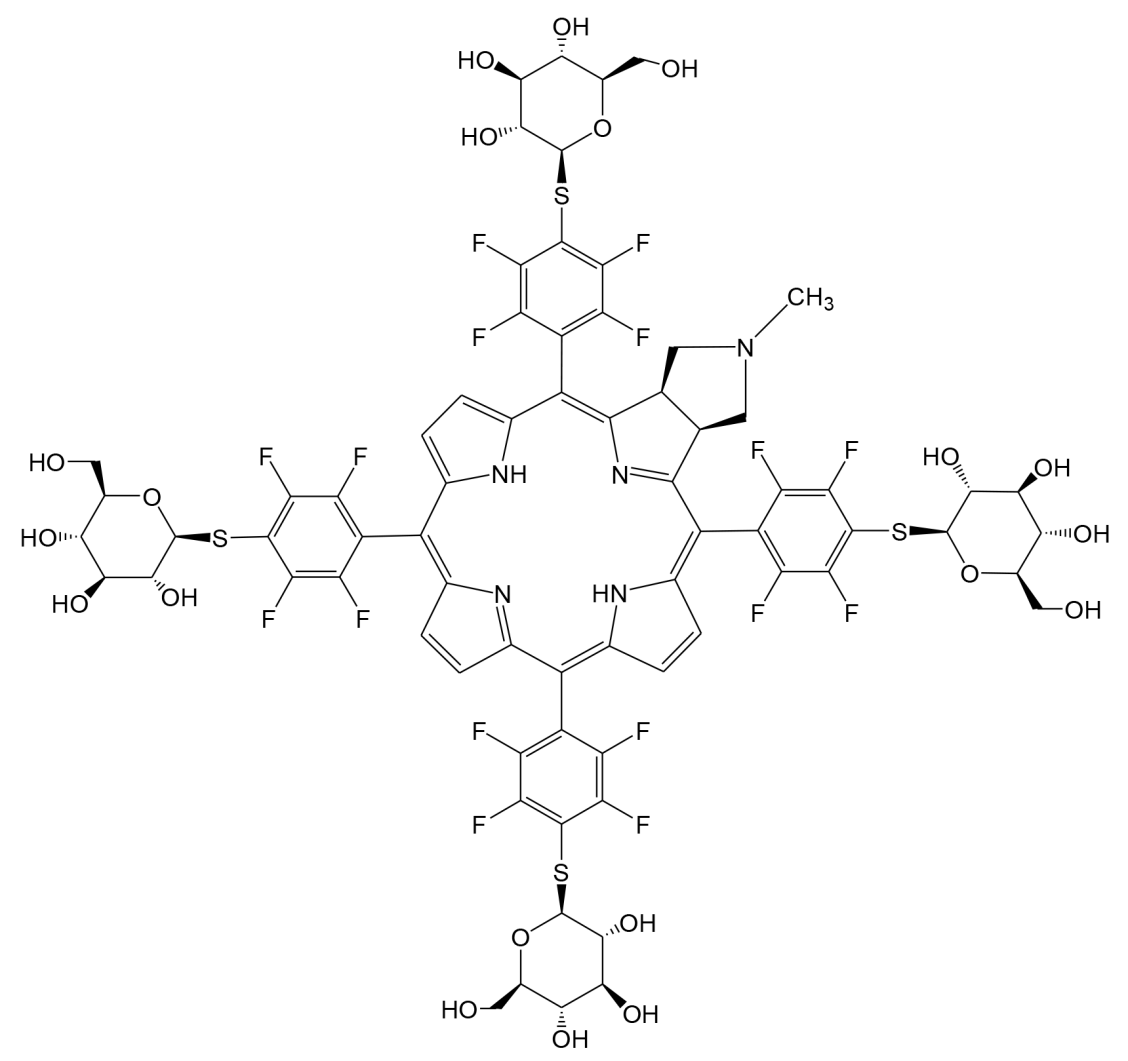
Chemical structure of G-chlorin Glycoconjugated chlorin; 5, 10, 15, 20-tetrakis (4-(β-D-glucopyranosylthio)-2, 3, 5, 6- tetrafluorophenyl)-2, 3-(methano (*N*-methyl) iminomethano) chlorine

G-chlorin (H_2_TFPC-SGlc [glycoconjugated chlorin; 5, 10, 15, 20-tetrakis (4-(β-D-glucopyranosylthio)-2, 3, 5, 6-tetrafluorophenyl)-2, 3-(methano (*N*-methyl) iminomethano) chlorine]) (Figure 6) was provided by laboratories of Kyoto University (Japan) and Okayama University of Science (Japan) [38]. Mitoxantrone (Novantrone®; dihydroxyanthracenedione) was purchased from ASKA Pharmaceutical Co., Ltd. (Tokyo, Japan).

### Cell culture

The mouse colon cancer cell line CT26 (American Type Culture Collection, No. CRL-2638) was cultured in RPMI1640 (Sigma-Aldrich, St. Louis, MO) supplemented with 10% fetal bovine serum (FBS) and 1% ampicillin and streptomycin. The human colon cancer cell lines HT29 (American Type Culture Collection, No. HTB-38) and HCT116 (American Type Culture Collection, No. CCL-247) were cultured in McCoy's 5A Medium (Sigma-Aldrich) supplemented with 10% FBS and 1% ampicillin and streptomycin. Cells were cultured in an atmosphere of 5% CO_2_ at 37°C.

### Animals and tumor models

Female wild-type (BALB/c) and female nude (BALB/c Slc-nu/nu) mice were obtained from Japan SLC (Kyoto, Japan). Animals were allowed to acclimatize for two weeks in the animal facility before any intervention was initiated. The procedures in these experiments were approved by the Nagoya City University Center for Experimental Animal Science, and mice were cared for according to guidelines of the Nagoya City University for Animal Experiments.

### *In vivo* PDT

The allograft or xenograft tumor models were established by subcutaneously implanting 1 × 10^6^ CT26 cells in 200 μL of PBS. Ten days after tumor inoculation, mice were administered an intravenous injection (via the tail vein) of G-chlorin at a dose of 1.25 μmol/kg. Four hours after the injection, the tumors were illuminated with 660-nm LED light (OptoCode Corporation, Tokyo, Japan) at a dose of 40 J/cm^2^ (intensity: 49 mW/cm^2^) at the skin immediately above the tumor. Tumor growth was monitored every day by measuring tumor volume with a vernier caliper. Tumor volume was calculated using the following formula: (length × width × depth)/2. Results were analyzed using Welch's *t*-test.

### *In vitro* PDT

Cells were incubated with G-chlorin in the culture medium for 4 hours. They were washed once with PBS, then the wells were filled with PBS, and the cells were irradiated with 16 J/cm^2^ (intensity: 30.8 mW/cm^2^) of 660-nm LED light. The PBS in the wells was replaced with medium supplemented with 2% FBS, and the cells were incubated for a specified time before analysis.

### Western blotting

Intracellular protein samples were collected using cell lysis buffer (Cell Signaling Technology, Beverly, MA). After disruption in an ice bath using a Bioruptor sonicator (Cosmo Bio Co., Ltd., Tokyo) for 15 seconds, lysates were centrifuged at 15,000 rpm for 10 minutes at 4°C. The cell membrane protein samples were collected using a Mem-PER eukaryotic membrane protein extraction reagent kit (Thermo Scientific, MA, US 89826) according to manufacturer instructions.

Each sample was normalized to an equal protein concentration using a protein assay kit (Bio-Rad Laboratories, Tokyo, Japan). An equal volume of 2X SDS-PAGE sample buffer was added to each sample, followed by boiling for 5 minutes at 100°C. Aliquots of the samples were fractioned on an 8% to 15% SDS-PAGE gel and were electroblotted onto a nitrocellulose membrane. The membrane was blocked with 5% skimmed milk in PBS (−) for 1 hour at room temperature. The membrane was then incubated with primary antibodies, anti-CRT (Santa Cruz Biotechnology, Inc., CA., 1:1000) or anti-HMGB1 (Chondrex, Redmond, WA; 1:1000), overnight at 4°C and was then washed with 0.05% Tween 20 in PBS (−) three times at 5-minute intervals. The membrane was incubated with secondary antibody for 1 hour at room temperature followed by three washes with 0.05% Tween 20 in PBS (−) at 5-minute intervals. Filters were stripped and re-probed with either a monoclonal β-actin antibody (Abcam, Tokyo, Japan) or a pan Cadherin antibody (Abcam) as an internal control.

### Immunofluorescence microscopy

Samples were fixed with ethanol and acetone. Incubation with primary antibodies against CRT (Santa Cruz Biotechnology) or HMGB1 (Chondrex) was carried out in a solution of PBS containing 0.1% milk. The secondary antibody was Alexa Fluor 488 goat anti-mouse IgG (H + L; Invitrogen, Tokyo, Japan). All sections were counterstained with 4′, 6-diamidino-2-phenylindole (DAPI) (Kirkegaard and Perry Laboratories). Images were obtained using a confocal laser microscope (Nikon A1 confocal system (Nikon Instech Co., Ltd., Tokyo, Japan)), and data were analyzed using NIS element imaging software (Nikon Instech). Band-pass emission filters of 405 nm and 488 nm were used.

### Immunohistochemistry

The tumors were immediately excised and fixed in formalin for immunohistochemical examination. Paraffin-embedded specimens were sectioned (4 μm), pre-treated with Bond Epitope Retrieval Solution 2 (Leica Biosystems Nussloch GmbH, Nussloch, Germany), and stained with anti-CRT polyclonal antibody (bs-5913R) (Bioss Inc., Woburn, MA, USA) (1:200) or anti-HMGB1 monoclonal antibody (ab79823) (Abcam plc, Cambridge, UK) (1:800), followed by staining with BOND-MAX (Leica Biosystems Nussloch GmbH) according to the manufacturer's instructions.

### Antitumor vaccination

CT26 cells were incubated with G-chlorin for 4 hours then irradiated with 16 J/cm^2^ of 660-nm LED light. Four hours after irradiation, 5 × 10^5^ CT26 cells treated with G-chlorin PDT in 200 mL PBS were inoculated subcutaneously into the lower flank of BALB/c mice. Untreated cells (1 × 10^6^) were inoculated into the contralateral flank 7 days later. The percentage of tumor-free mice in each group was calculated using Kaplan-Meier survival analysis, and the log-rank statistic was used to compare the curves between groups.

### Small interfering RNA transfections

CT26 cells were transfected with small interfering RNA (siRNA) for either CRT or HMGB1 (Ambion, Beverly, MA) using Lipofectamine reagent (Invitrogen) according to manufacturer instructions. At 48 hours after transfection, CT26 cells were assessed for total content of each protein by western blotting.

### Recombinant protein

Cells were exposed to recombinant CRT (MyBioSource, San Diego, CA) at 3 mg per 10^6^ cells in phosphate buffered saline (PBS) on ice for 30 minutes. Recombinant HMGB1 (Sino Biological Inc. Beijing, China) at 200 ng per mouse was injected along with dying tumor cells.

### Statistical analysis

Descriptive statistics and simple analyses were carried out using the statistical package R version 3.1.2 (www.r-project.org/). In all analyses, a P-value < 0.05 was considered statistically significant

## SUPPLEMENTARY FIGURES


