# Supplementary material for: Immunogenic cell death due to a new photodynamic therapy (PDT) with glycoconjugated chlorin (G-chlorin)
Source: Oncotarget. 2016 May 30;7(30):47242–51. doi: 10.18632/oncotarget.9725 (PMC5216938; doi:10.18632/oncotarget.9725)
Supplement: Supplementary file 1 [file oncotarget-07-47242-s001.pdf]

Immunogenic cell death due to a new photodynamic therapy (PDT) with glycoconjugated chlorin (G-chlorin)

SUPPLEMENTARY FIGURES

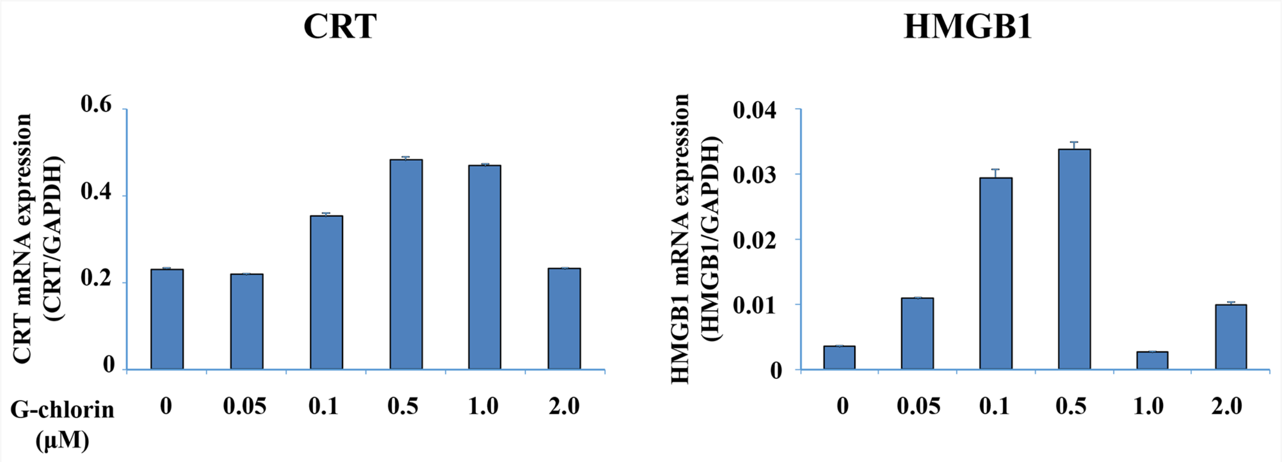

Supplementary Figure S1: Expression of mRNA of CRT and HMGB1 by PDT. CT26 cells were treated with G-chlorin for 4 hours and then irradiated with 16 J/cm2 of 660-nm LED light. The CRT and HMGB1 mRNA expression levels were measured by real-time PCR immediately after the 4-hour treatment. Data are the means of three independent experiments ± SD.

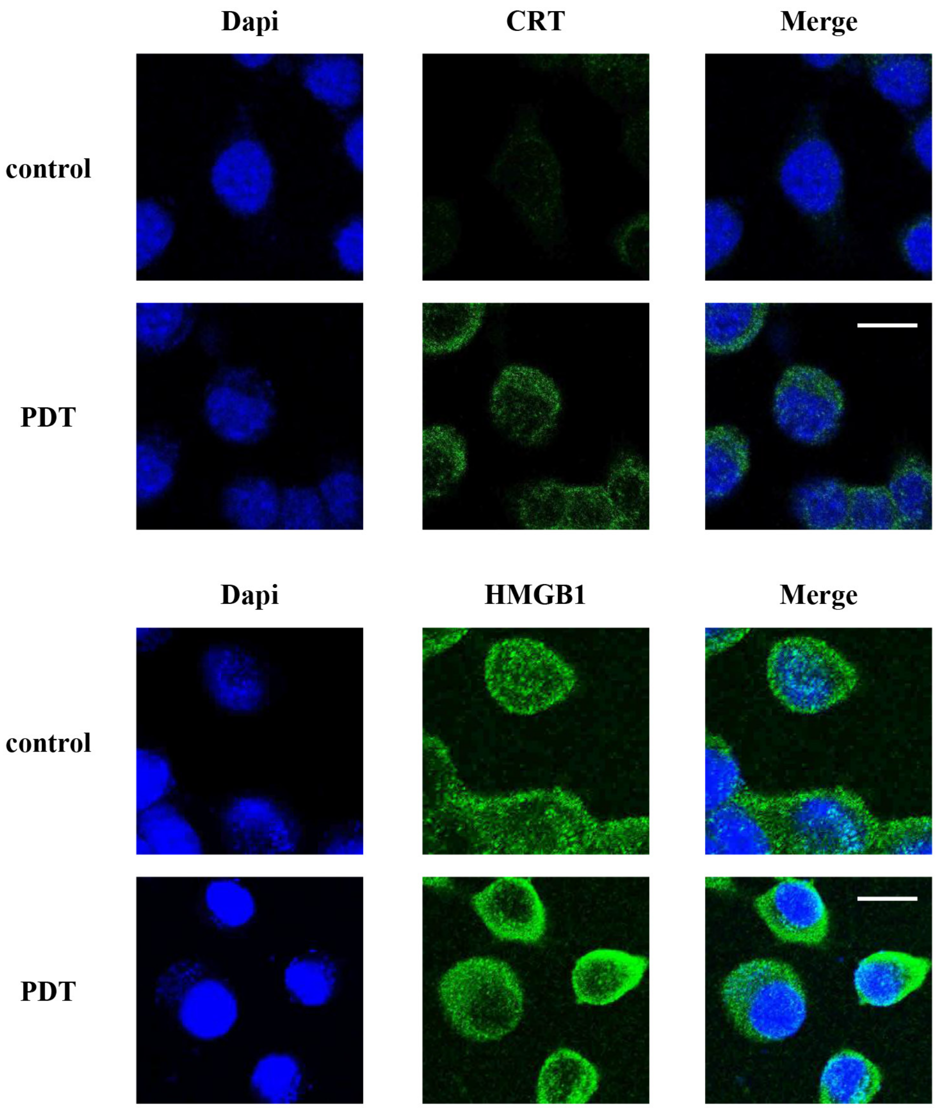

Supplementary Figure S2: Translocation of CRT and HMGB1 by PDT at lower magnification. CT26 cells were treated with G-chlorin for 4 hours and irradiated with 16 J/cm2 of 660-nm LED light. Translocation of CRT and HMGB1 was assessed by immunofluorescence staining at 4 hours after treatment immediately after the 4-hour treatment. Images were obtained using confocal microscopy (original magnification ×600; scale bar = 10 µm).
